# Nature-based experience in Venetian lagoon: Effects on craving and wellbeing in addict residential inpatients

**DOI:** 10.3389/fpsyg.2024.1356446

**Published:** 2024-06-12

**Authors:** Giulia Benvegnù, Mauro Semenzato, Alberto Urbani, Isadora Zanlorenzi, Mauro Cibin, Cristiano Chiamulera

**Affiliations:** ^1^Department of Diagnostics and Public Health, University of Verona, Verona, Italy; ^2^Centro Soranzo, Mestre, Italy

**Keywords:** nature experience, craving, substance use disorders, residential programs, wellbeing

## Abstract

**Introduction:**

It is known that exposure to the natural environment may positively modulate mental processes and behaviors; in particular, it can reduce stress, anxiety, and depressive symptoms. This suggests a potential integration of “nature experience” into the treatment for substance use disorder (SUD) since various types of addiction are associated with anxiety and depression. Considering that only one study has been reported to date in patients with alcohol use disorder, the effect of nature experience in SUD patients' needs to be further investigated. This study aimed to test the effects of exposure to a natural lagoon environment on craving and measures of wellbeing in SUD patients in comparison to exposure to an urban environment.

**Methods:**

Twenty-four SUD patients were divided into three groups of eight participants and exposed to two walking sessions (interspersed with a 1-week wash-out period) in a natural environment typical of the Venetian lagoon, an Urban walk, or staying at the residential center based on a Latin-square design. Before and after each session, drug craving, mood, wellbeing, agency, openness to the future, and restorativeness were assessed.

**Results:**

The Nature walk significantly decreased craving in participants compared to their pre-walk values, and compared to craving after the Urban walk, with the latter significantly increased vs. pre-walk values. The Nature walk significantly decreased negative mood and increased wellbeing and agency. Openness to the future and restorativeness measures showed significant improvement after the Nature walk compared to the Urban walk. On the other hand, craving scores after the Urban Walk positively correlated with negative mood and a Sense of Negative Agency values and negatively correlated with wellbeing scores.

**Discussion:**

Our results confirm that “nature experience” may improve mood, wellbeing, attention, stress relief, openness, and sense of being active in SUD patients. Moreover, we also showed a specific effect on drug craving—a key symptom of SUD.

## 1 Introduction

It is widely known that the environmental context may play an important role in the modulation of mental processes and behaviors. The physical features of built and natural spaces have been shown to affect the brain mechanisms of mental health and disorders, thus potentially having a beneficial impact on behavior and health outcomes (Hartig and Kahn, 2016). Specifically, observational and experimental studies have demonstrated that contact with nature (“nature experience”) may induce acute, and sometimes persistent (Bratman et al., 2012, Supplementary material), effects on several physiological and psychological measures such as improvement of mood, attention, and reduced arousal (Hartig et al., 2011; Bratman et al., 2012; Kuo, 2015).

The biophilia hypothesis suggests that the human brain is evolutionarily wired to recognize and adapt to the natural (or nature related) environments. Nature-related features of the environment signal positive reinforcement and positive emotional effects. This phenotype owned an evident survival advantage in the past, and it is supposed to be still present in modern *Homo sapiens* (Wilson, 1984). This hypothesis is best supported by the vast evidence of mental benefits and improved wellbeing experienced after exposure to natural environments, plants, and nature views. Among the different theories supporting the effect of nature experience, the “Attention Restoration Theory” suggests that time spent in green areas restores cognitive processes through the practice of effortless attention (Kaplan and Kaplan, 1989; Kaplan, 1995; Berto, 2005; Ohly et al., 2016). The “Stress Reduction Theory” or “Psychoevolutionary Theory” proposes that contact with nature reduces stress and normalizes cardiovascular parameters controlled by the sympathetic system through an ‘*automatic positive affective response*” (Ulrich et al., 1991). More recent studies showed that exposure to nature may facilitate the regulation of emotions by activating the parasympathetic system (Richardson et al., 2016).

“Forest bathing” (aka Shinrin-Yoku) is an exemplary practice of contact with nature consisting of (guided or not) forest walking that promotes mental and physical wellbeing by acting on physiological parameters (e.g., heart rate, blood pressure), cognition, and affective and mood states, as well as on healthy behaviors (Hansen et al., 2017; Kobayashi et al., 2018; Twohig-Bennett and Jones, 2018; Corazon et al., 2019; Kotera et al., 2021). The beneficial effects of “forest bathing” on the reduction of stress and depression suggest a rationale for potential integration into therapeutic interventions for addiction (Kotera and Rhodes, 2020; Kotera et al., 2022; Yi et al., 2022) based on the relevant component of affective and mood disturbances in substance use (Smith and Book, 2008) and non-pathological gambling (Medeiros et al., 2016) disorders. To our knowledge, only one study assessed the effects of nature experience in people with a diagnosis of alcohol use disorders, showing significant improvement in depressive symptoms (Shin et al., 2012). Similarly, a study on patients with gambling disorder found that even just listening to high-definition forest sounds on headphones decreased negative mood compared to city sounds (Ochiai et al., 2020).

Therefore, it is important to understand whether the proven efficacy of nature-based intervention for different mental disorders may be of benefit for addiction treatment. In a study by Tesler et al., a sample of at-risk adolescents (but with no SUD diagnosis) after three activity sessions in a forest showed an increase in perceived wellbeing and a decrease in psychosomatic symptoms and tobacco/alcohol use (Tesler et al., 2018). Despite the lack of studies that have tested the effects of direct exposure to natural environments in SUD patients, some other studies suggest an effect on craving—the emotionally charged mental state characterized by the desire to take the substance(s) of abuse (May et al., 2015). For example, some studies has used imagery-based tasks involving natural settings to help reduce cravings for nicotine and food (e.g., Versland and Rosenberg, 2007; Hamilton et al., 2013). The online cross-sectional survey by Martin et al. found that access to a garden or allotment was associated with a reduction in the frequency and strength of craving (for alcohol, nicotine, and caffeine). Residential neighborhoods incorporating more than 25% green space were correlated with similar reductions in craving. Interestingly, these effects were mediated by a decrease in negative mood (Martin et al., 2019).

On this basis, the present research aims, as a first objective, to investigate the effects of exposure to nature compared to an urban environment on craving, mood, and wellbeing in a sample of SUD patients. Second, it aims to test the effects of such exposure on the sense of agency (i.e., the capacity to claim authorship over an action and associate specific consequences with a specific action; Hurault et al., 2020) and openness to the future (i.e., having positive expectations and a general disposition of acceptance toward the future; Colombo et al., 2020). Considering that these constructs have been shown to be protective factors for mental health and positively associated with wellbeing (Weinstein, 1980; Moore, 2016; Botella et al., 2018; Mikus et al., 2018; Colombo et al., 2020), we aimed to assess whether the effects of nature exposure on craving were correlated to the effect of these measures. We also assessed the effect of the nature vs. urban exposure sessions on restorativeness (i.e., physical, spatial, and non-spatial features of a setting that contributes to the recovery of psychological equilibrium; Kaplan, 1995).

A lagoon environment (Venice nature hinterland) was chosen as nature exposure in the present research. Considering that addiction treatment services are often located in urban areas, contact with forests or mountains might not be feasible. On the other hand, it would be important to assess the potential beneficial effects of nature experience in nearby natural environments, especially in the case of residential addiction centers located in extra-urban areas. Moreover, studies in different types of natural environments other than forests/mountains are needed to better understand the effects of a broader spectrum of nature experience.

In this study, we exposed a group of addict inpatients at the residential facility “Centro Soranzo” (Venice, Italy) to two 1-h walks in the typical natural environment of the Venetian lagoon and, after a 1-week wash-out period to an Urban walk session (in Mestre, mainland borough of the Venice municipality). The measures of craving, mood, wellbeing, openness to the future, agency, and restorativeness were collected before and after each walking session to assess the effects of nature vs. urban experiences. Particularly, we hypothesize to find a reduction in craving (H1) and negative mood (H2) and an increase in wellbeing (H3), openness to the future (H4), and a sense of agency (H5) after exposure to a natural environment compared to the urban environment. We, therefore, expect the natural environment to be perceived as more restorative than the urban environment (H6). Finally, we hypothesize an association between craving and negative mood (H7) and consequently that higher levels of craving are associated with lower levels of perceived wellbeing (H8), openness to the future (H9), and agency (H10).

## 2 Materials and methods

### 2.1 Participants

The experimental sample consisted of patients (age 18–65 years, *M* = 37.79, SD = 8.08) of both sexes, who are native Italian speakers, with a diagnosis of pathological addiction under treatment at the “Centro Soranzo” residential center (Venice, Italy). All participants received the Treatment-As-Usual (TAU) of the residential facility program for drug detoxification and rehabilitation, which includes interventions for addiction (relapse prevention group therapy and anti-craving pharmacotherapy) and individual and group psychotherapy by using verbal and non-verbal techniques (e.g., psychosomatic training, mindfulness, yoga, art therapy, expressive laboratory, dance-movement therapy, pet therapy, song therapy, autogenic training, and psycho-educational activities). It may also be possible for them to attend Alcoholics Anonymous (AA) group therapy. Comorbid psychiatric patients with anxiety or depression symptoms were supported by psychiatric intervention by using the appropriate pharmacotherapy if necessary. Pharmacotherapies prescribed *ad hoc* at the center for the treatment of anxiety/depressive center symptoms included antidepressants, anxiolytics, antipsychotics, lithium, mood stabilizers, and melatonin.

The presence of organic diseases that made prolonged walking difficult was used as an exclusion criterion. Twenty-four male patients (male = 20) took part in the study and were segregated into three groups through a semi-randomized distribution. Participants were admitted to the residential facility for an average of 1 month before the start of the study. The diagnosis of pathological addiction was made by territorial addiction services (SerDs). One-third of them have comorbidity with other disorders, especially mood disorders. They did not have a long history of addiction treatment failures (ranging from no previous residential treatment to a maximum of two previous experiences in other facilities). The demographics of the participants are shown in Table 1.

**Table 1:** Demographic data of the three groups.

	**Group 1 (*N* = 8)**	**Group 2 (*N* = 8)**	**Group 3 (*N* = 8)**
Age (*M* ± SD)	39.00 ± 9.57	39.00 ± 6.67	35.37 ± 8.27
Gender (men/women)	8/1	7/2	8/1
Education (MS/HS/BSc/MSc)	3/5/0/0	1/4/0/2	0/6/2/0
Primary addiction (C/H/A/G/B)	6/1/0/1/0	3/0/3/1/1	5/1/2/0/0

MS, Middle school; HS, High school; BSc, Bachelor of Science; MSc, Master of Science; C, cocaine; H, heroin; A, alcohol; G, gambling; B, benzodiazepines.

The study was approved by the Scientific Board of the “Centro Soranzo” (18/04/2023 meeting; no internal protocol study number is available). The research complies with the guidelines for human studies and was conducted ethically by the World Medical Association's Declaration of Helsinki.

### 2.2 Measures

We measured craving, mood, wellbeing, openness to the future, agency, and restorativeness. The questionnaires used are given below.

- An *ad-hoc* questionnaire for measuring craving for the substance(s) of abuse. A single-item (“How much craving do you feel right now?”), 10-point scale based on the literature and adapted from the studies of Traylor et al. (2011) and Benvegnù et al. (2021) was adopted.
- Profile of Mood States (POMS) (McNair et al., 1971; italian adaptation: Farnè et al., 1991). The questionnaire consists of a list of 58 adjectives, with a 5-point scale response, measuring six factors: Tension/Anxiety (T; a state of intensified physical tension caused by anxiety and described by words of “Nervous” or “Restless”), Depression/Dejection (D; self-inadequacy hallmarked by sadness, guilt, loneliness, and hopelessness), Anger/Hostility (A; a feeling of anguish and hostility described by words such as “Annoyed” or “Resentful”), Vigor/Activity (V; a cheerful mood referring to high, positive energy described by words such as “Lively” or “Active”), Fatigue/Inertia (S; a state of low energy and described by words of “Exhausted” or “Weary”), and Confusion/ Bewilderment (C; a state of cognitive inefficiency described by words like “Forgetful” or “Bewildered”). The total score [total mood disturbance (TMD)] was calculated as the sum of the four negative scales (T, D, A, and S) minus the positive one (V). Cronbach's alpha of the total score is 0.95.
- Flourishing Scale (FS) (Diener et al., 2010; italian validation: Giuntoli et al., 2017): An 8-item scale with a 5-point Likert scale response provides a single psychological wellbeing score given by the sum of all items. The construct measured by the FS can be called “eudaimonic wellbeing” and summarizes dimensions that describe individuals' optimal psychological functioning (e.g., meaning in life, positive relationships, and self-acceptance measured by items such as “My social relationships are supportive and rewarding”). Cronbach's alpha is 0.85.
- Openness to the Future Scale (OFS) (Botella et al., 2018): OFS is a 10-item scale with a 5-point Likert scale response. “Openness to the future” refers to the active process of accepting future scenarios and trusting in one's personal ability to make plans to achieve desired outcomes and cope with adversity. It is measured with items such as “When I make plans, I am sure I will be able to carry them out.” Total openness to the future score is calculated as the sum of all items. Cronbach's alpha is 0.82.
- Sense of Agency Scale (SoAS) (Tapal et al., 2017): This scale is a French adaptation (Hurault et al., 2020) of the original SoAS involving 7 items with a 7-point Likert scale response. It measures two constructs: Sense of Positive Agency (SoPA, which represents the level of control over the body, mind, and environment felt by an individual and is measured by items such as “I am in full control of what I do”) and Sense of Negative Agency (SoNA, which represents the lack of control over the body, mind, and environment and the perception of being existentially helpless. It is described by items such as “My actions just happen without my intention”). Cronbach's alpha value is 0.82 for SoPA and 0.53 for SoNA.
- Perceived Restorativeness Scale (PRS-11) (Hartig et al., 1997; italian validation of the short-form: Pasini et al., 2014): The Italian validation includes 11 items (e.g., “To stop thinking about the things that I must get done I like to go to places like this”) with an 11-point response scale. It measures four factors: Fascination (referring to the effortless and involuntary attention to the natural setting that is the key process to restoring from mental fatigue), Being away (referring to escaping from unwanted distractions in the surroundings and distancing oneself from one's usual work), Coherence (referring to a physically coherent environment that sustains exploration; the perception of the environment as a whole with a larger organizational structure), and Scope (referring to the environmental characteristics that extend in time and space so that the environment is perceived to be possible to enter and spend time in). Cronbach's alpha of the total score is 0.82.

### 2.3 Procedure

All participants, after signing an informed consent and checking their adherence to the inclusion/exclusion criteria, were asked to complete a short demographic questionnaire. They were then semi-randomly allocated into three eight-participant groups to be exposed according to a Latin square design to two walking sessions in nature or staying at the center (interspersed with a 1-week wash-out period). All participants underwent the three conditions (two walking sessions and one “staying session”). The two walking sessions took place (i) in an urban environment (in Mestre, a mainland borough of the Venice municipality; henceforth “City condition”), and (ii) in a natural environment typical of the Venice lagoon (natural hinterland of Venice; henceforth “Nature condition”).

The lagoon environment includes wetlands and salt marshes characterized by the presence of trees, shrubs, reeds, rushes, sedges, and salt-tolerant coastal vegetation, such as seagrass and glasswort. A typical feature is the “*barena*” (mudflat)—sandy areas covered and uncovered by the tides.

In contrast, the urban environment includes tree-lined boulevards with moderate car traffic, bordered on both sides by residential buildings averaging 4–5 stores high, and areas with predominantly commercial establishments, offices, restaurants, and parking lots. The Urban walk took place along a loop route in the city's most architecturally valuable areas but to avoid both the most degraded and drug-associated areas.

**Figure 1:**
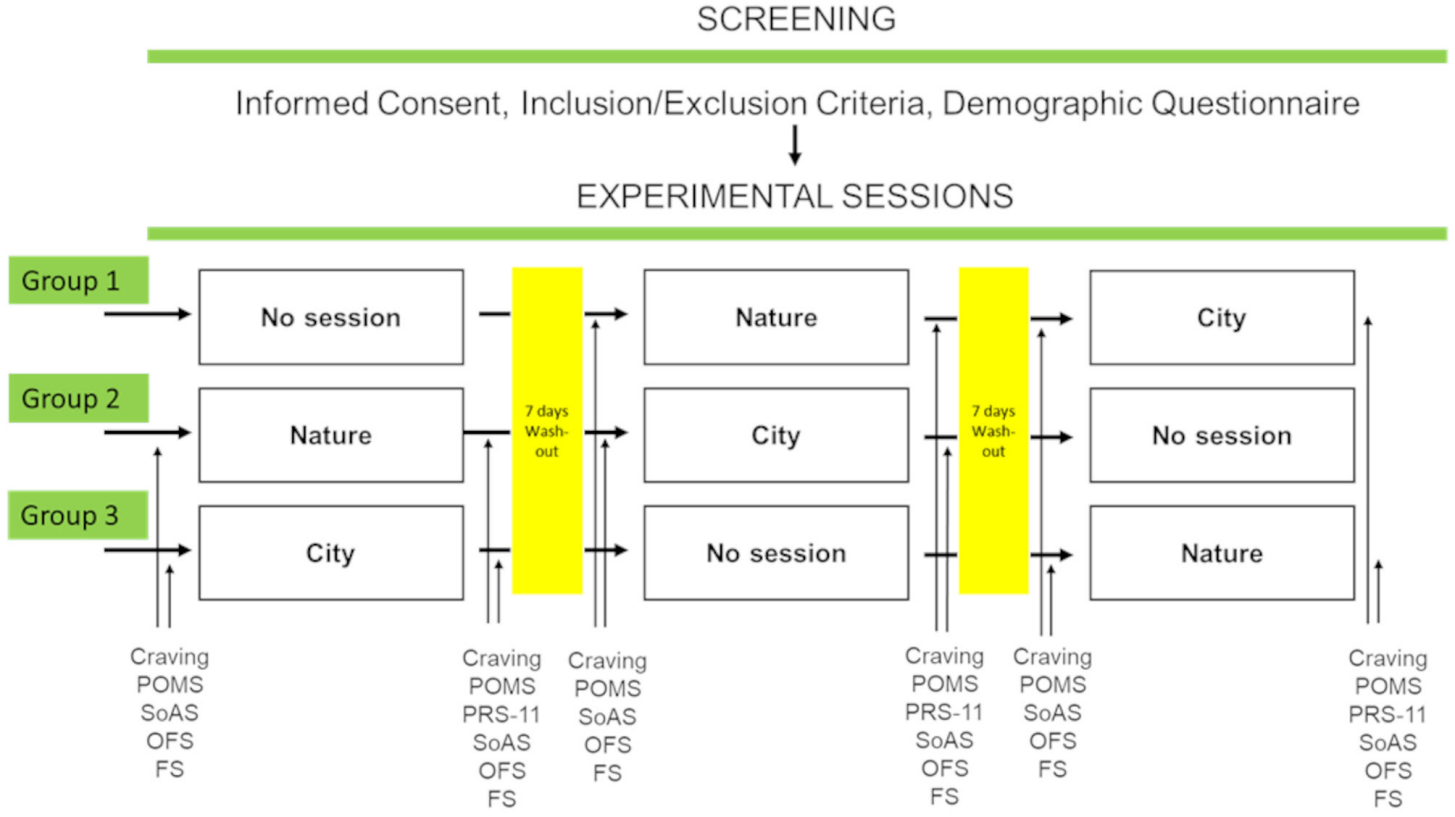
Schematic representation of the phases of the procedure based on a Latin-square design. POMS, Profile of Mood States; SoAS, Sense of Agency Scale; OFS, Openness to the Future Scale; FS, Flourishing Scale; and PRS-11, Perceived Restorativeness Scale.

During the “staying session,” and during the entire period of the study, participants performed unstructured activities at the residential facility and received the TAU (Figure 1). No data were collected during the session and time at the center.

During both City and Nature conditions, participants took an easy, no-slope 1-h walk (tourist difficulty) led by a practitioner and were free to interact with each other and contemplate the view. Before and after each walking session, participants completed the *ad-hoc* craving measurement questionnaire, POMS, SoAS, OFS, and FS. At the end of each session, the PRS-11 was completed.

### 2.4 Data analysis

All analyses were performed with GraphPad Prism 9.1.0 and Microsoft Excel (Version 2404 Build 16.0.17531.20140). To verify H1, a two-way ANOVA (mixed-effects analysis) with Greenhouse– Geisser correction was performed on craving scores, with “CONDITION” as the between factor (two levels: “City” and “Nature”) and “TIME” as the within factor (two levels: “pre-” and “post-” session). In case of significance, *Post-hoc* comparisons were conducted (Fisher's LSD test). To test hypotheses H2–H5, the same analyses were conducted for POMS scores (H2) – for both Total Mood Disturbance (TMD) and subscales –, FS scores (H3), OFS scores (H4), and SoAS scores (H5)—for both Sense of Positive Agency (SoPA) and Sense of Negative Agency (SoNA). The subscales of PRS-11 and the total scores were analyzed by paired *t*-test (or Wilcoxon signed-rank tests in case of non-normal data) to test H6.

To verify hypotheses H7–H10, correlations between craving scores and POMS scores (H7), FS scores (H8), OFS scores (H9), and SoAS scores (H10) were tested with Spearman's rank correlation coefficient ρ.

Descriptive statistics of the instruments used in the three groups for each condition and time point are reported in Supplementary Table S1.

## 3 Results

### 3.1 Craving

**Figure 2:**
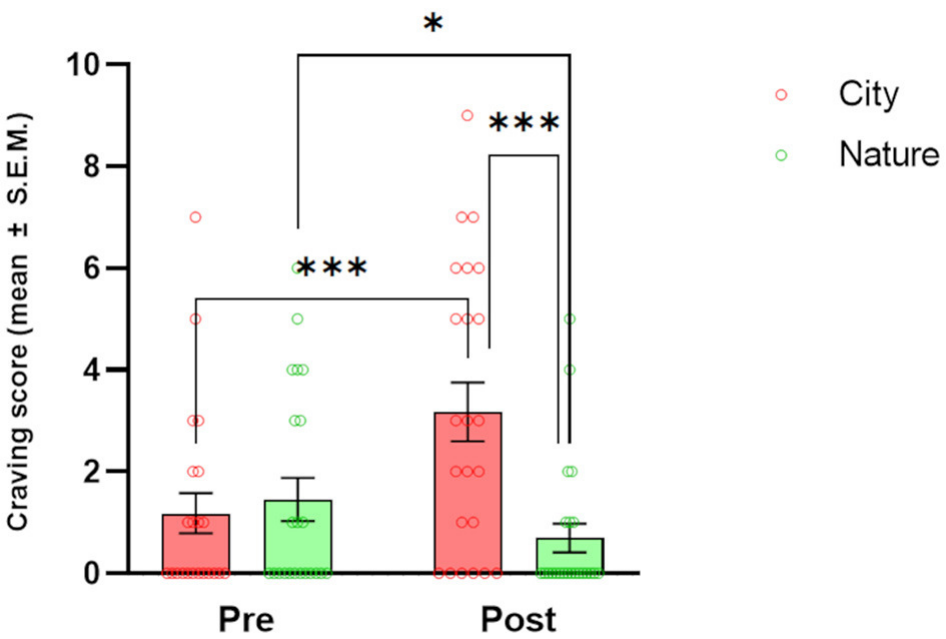
Effects of natural and urban environments on craving scores. The bars represent the mean score (± SEM) of craving measured at the beginning of the session (pre) and after the walking session (post) in the lagoon (Nature; green markers) and in the urban environment (City; red markers). *p < 0.05 and ***p < 0.001, Fisher's LSD *Post-hoc* test.

There was a significant CONDITION [*F*_(1, 23)_ = 17.45, *p* < 0.001] main effect and CONDITION × TIME interaction [*F*_(1, 17)_ = 18.43, *p* < 0.001] for craving (Figure 2). *Post-hoc* tests showed a significant increase in craving from pre- to post-session in the City condition (*p* < 0.001) and an opposite effect in the Nature condition (*p* = 0.039). When the two conditions were directly compared, significantly higher craving was reported after the City than Nature condition at the post-session time point (*p* < 0.001).

### 3.2 Mood

**Figure 3:**
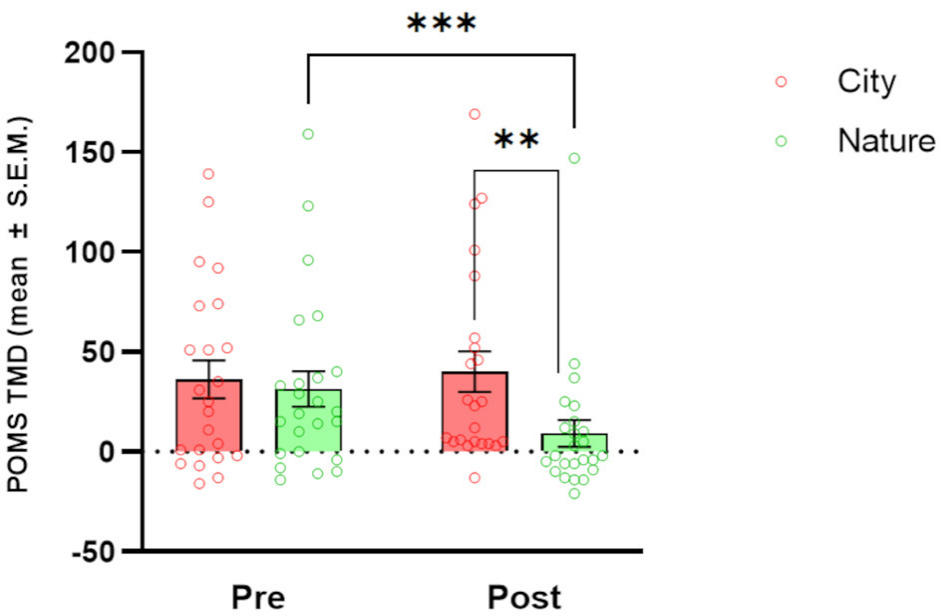
Effects of natural and urban environments on Profile of Mood States (POMS) scores. The bars represent the mean score (± SEM) of Total Mood Disturbance (TMD) measured at the beginning of the session (pre) and after the walking session (post) in the lagoon (Nature; green markers) and in the urban environment (City; red markers). **p < 0.01 and ***p < 0.001, Fisher's LSD *Post-hoc* test.

Analysis of POMS (Figure 3) showed significant main effects of TIME [*F*_(1, 23)_ = 4.98, *p* = 0.036] and CONDITION [*F*_(1, 23)_ = 6.07, *p* = 0.022] and a significant CONDITION × TIME interaction [*F*_(1, 21)_ = 10.13, *p* = 0.004]. *Post-hoc* tests showed a decrease in TMD values between the start and end of the session in Nature condition only [*p* < 0.001]. When the two conditions were directly compared, a significantly higher TMD in the City condition than in the Nature condition was observed after the post-session time point (*p* = 0.004).

### 3.3 Wellbeing

**Figure 4:**
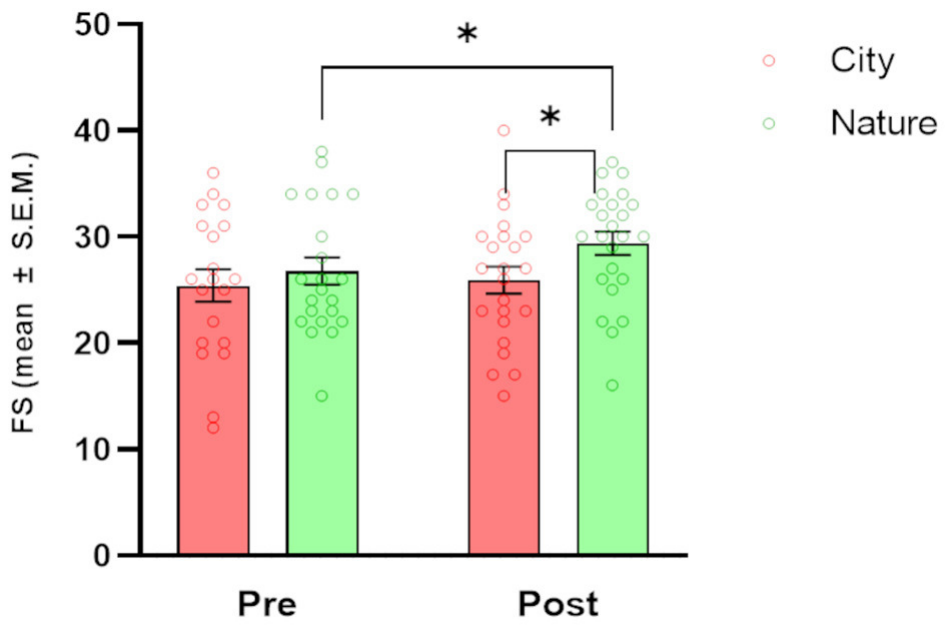
Effects of natural and urban environments on Flourishing Scale (FS) scores. The bars represent the mean score (± S.E.M.) of FS measured at the beginning of the session (pre) and after the walking session (post) in the lagoon (nature; green markers) and in the urban environment (city; red markers). *p < 0.05, Fisher's LSD *Post-hoc* test.

Regarding wellbeing (Figure 4), the FS showed significant main effects of TIME [*F*_(1, 23)_ = 4.66, *p* = 0.041] and CONDITION [*F*_(1, 23)_ = 5.02, *p* = 0.035]. *Post-hoc* tests showed a significant increase in FS scores from pre-session to post-session in the Nature condition only (*p* = 0.011). When the two conditions were directly compared, there were significantly higher FS scores in the Nature condition compared to the City condition at the post-session time point (*p* = 0.011).

### 3.4 Openness to the future

**Figure 5:**
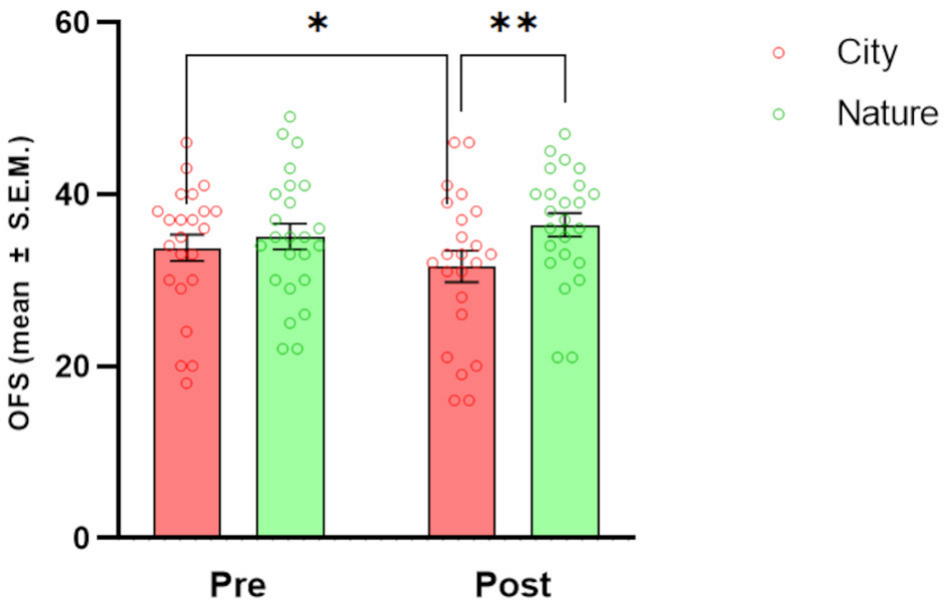
Effects of natural and urban environments on Sense of Positive Agency (SoPA) scores. The bars represent the mean score (± S.E.M.) of SoPA measured at the beginning of the session (pre) and after the walking session (post) in the lagoon (Nature; green markers) and in the urban environment (City; red markers). *p < 0.05 and **p < 0.01, Fisher's LSD *Post-hoc* test.

Analysis of the Openness to the Future scale (Figure 5) highlighted a significant CONDITION × TIME interaction [*F*_(1, 21)_ = 5.97, *p* = 0.023]. *Post-hoc* tests showed a significant decrease in OFS scores from pre-session to post-session in the City condition (*p* = 0.044). When the two conditions were directly compared, there was a significantly higher OFS score in the Nature condition compared to the City condition at the post-session time point (*p* = 0.007).

### 3.5 Sense of agency

**Figure 6:**
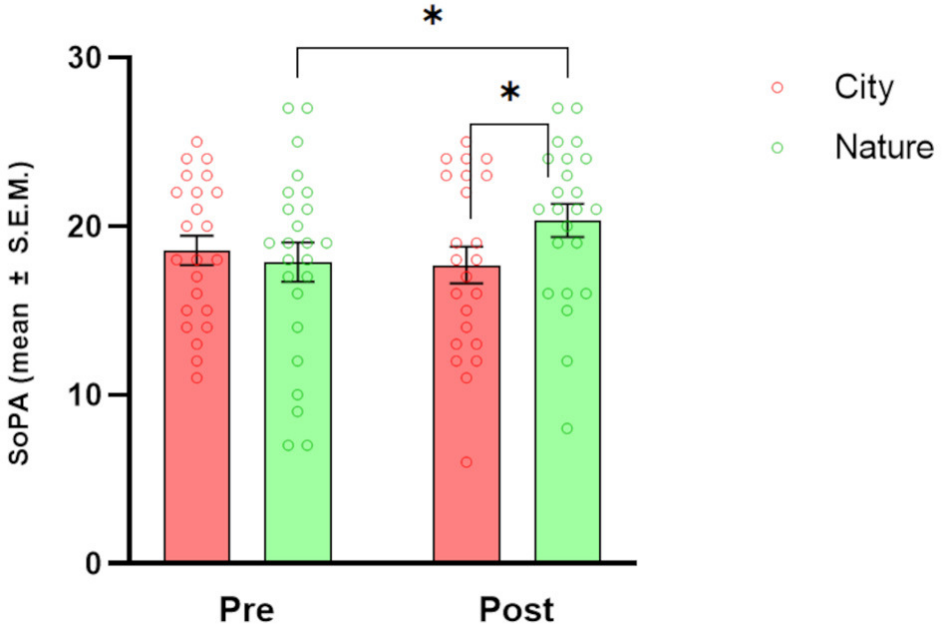
Effects of natural and urban environments on Openness to the Future Scale (OFS) scores. The bars represent the mean score (± S.E.M.) of OFS measured at the beginning of the session (pre) and after the walking session (post) in the lagoon (Nature; green markers) and in the urban environment (City; red markers). *p < 0.05, Fisher's LSD *Post-hoc* test.

Regarding Sense of Agency (Figure 6), the SoPA subscale showed a significant CONDITION × TIME interaction [*F* (1, 20) = 4.69, *p* = 0.042]. *Post-hoc* tests showed a significant increase in SoPA scores from pre-session to post-session in the Nature condition only (*p* = 0.042). When the two conditions were directly compared, there was a significantly higher SoPA score in the Nature compared to the City condition at the post-session timepoint (*p* = 0.046). Regarding the SoNA subscale, although the CONDITION × TIME interaction was significant [*F*_(1, 20)_ = 5.41, *p* = 0.031], *Post-hoc* tests did not reveal any significant differences.

### 3.6 Restorativeness

**Figure 7:**
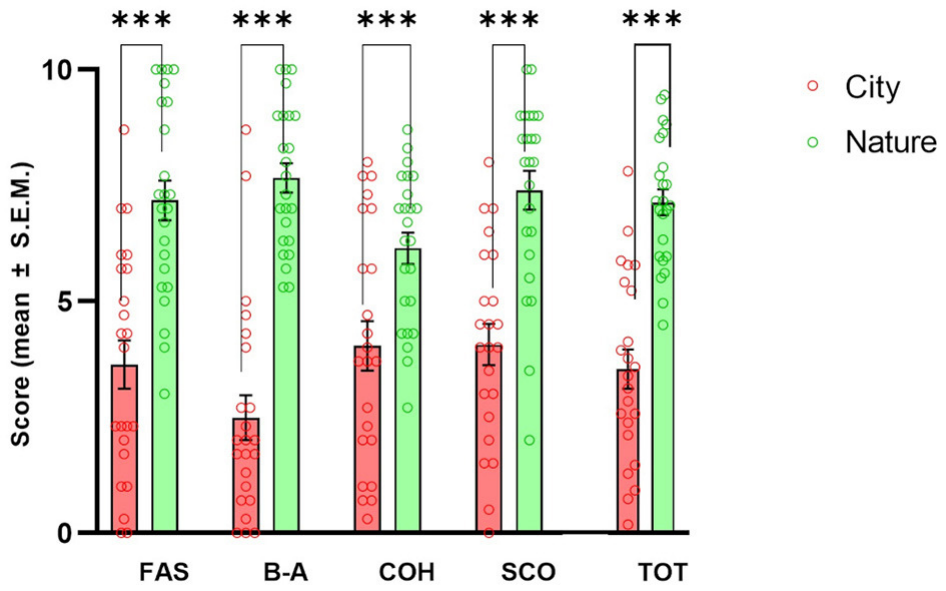
Effects of natural and urban environments on Perceived Restorativeness Scale (PRS-11) total score and factors. The bars represent the mean score (± S.E.M.) of Fascination (FAS), Being Away (B-A), Coherence (COH), Scope (SCO), and total score (TOT) measured after the walking session in the lagoon (Nature; green markers) and in the urban environment (City; red markers). ***p < 0.001, Fisher's LSD *Post-hoc* test.

All PRS-11 factors and the total score (Figure 7) were found to score significantly higher in the Nature condition than in the City condition: Fascination [*t*_(22)_ = 4.81, *p* < 0.001]; Being Away [*t*_(22)_ = 7.76, *p* < 0.001]; Coherence [*t*_(22)_ = 4.20, *p* < 0.001], Scope [*t*_(22)_ = 4.50, *p* < 0.001], and total score [*t*_(22)_ = 6.51, *p* < 0.001].

### 3.7 Association between craving and mood

**Figure 8:**
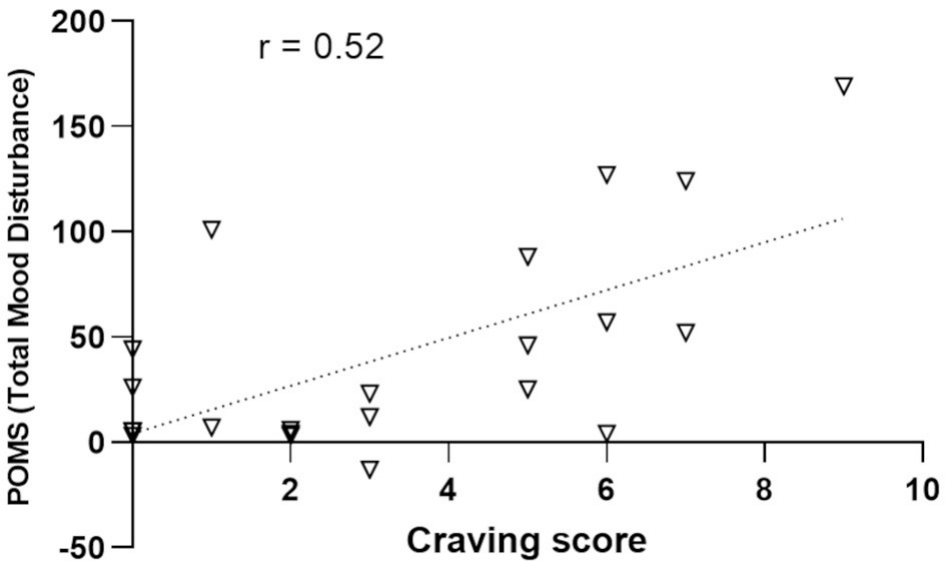
Correlational analysis between the craving scores measured after the walking session in the urban environment (abscissa) and the POMS global score (Total Mood Disturbance; ordinates).

The correlational analysis shows a series of significant positive correlations between craving scores measured at the post-session time point in the City condition and the POMS global score (Figure 8) and all subscales (except Vigor): Total Mood Disturbance: ρ = 0.52, *p* = 0.009; Tension: ρ = 0.72, *p* < 0.001; Depression: ρ = 0.44, *p* = 0.032; Anger: ρ = 0.49, *p* = 0.017; Fatigue: ρ = 0.60, *p* = 0.002; and Confusion: ρ = 0.48, *p* = 0.02. On the other hand, no significant correlation emerged between craving and POMS scores in the Nature condition.

### 3.8 Association between craving and wellbeing

**Figure 9:**
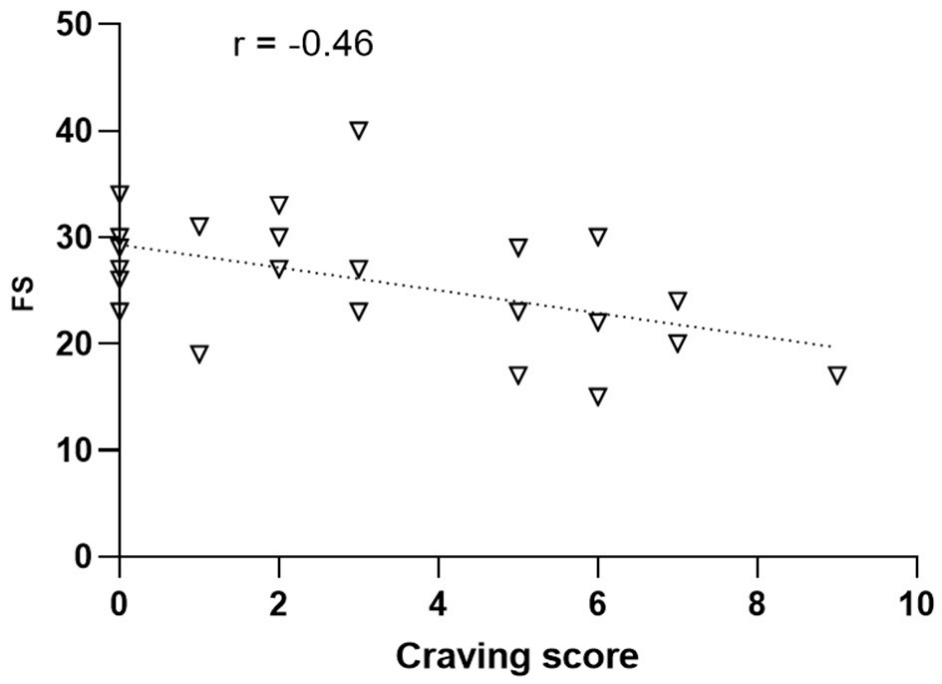
Correlational analysis between the craving scores measured after the walking session in the urban environment (abscissa) and the Flourishing Scale (FS; ordinates).

The correlation between the craving scores measured at the post-session time point in the City condition and wellbeing was significant (Figure 9). In particular, there was a negative correlation between craving and the FS scores (ρ = −0.46, *p* = 0.025). No significant correlations emerged in the Nature condition.

### 3.9 Association between craving and openness to the future

No significant correlations emerged between craving and OFS scores in both the Nature and City conditions.

### 3.10 Association between craving and sense of agency

**Figure 10:**
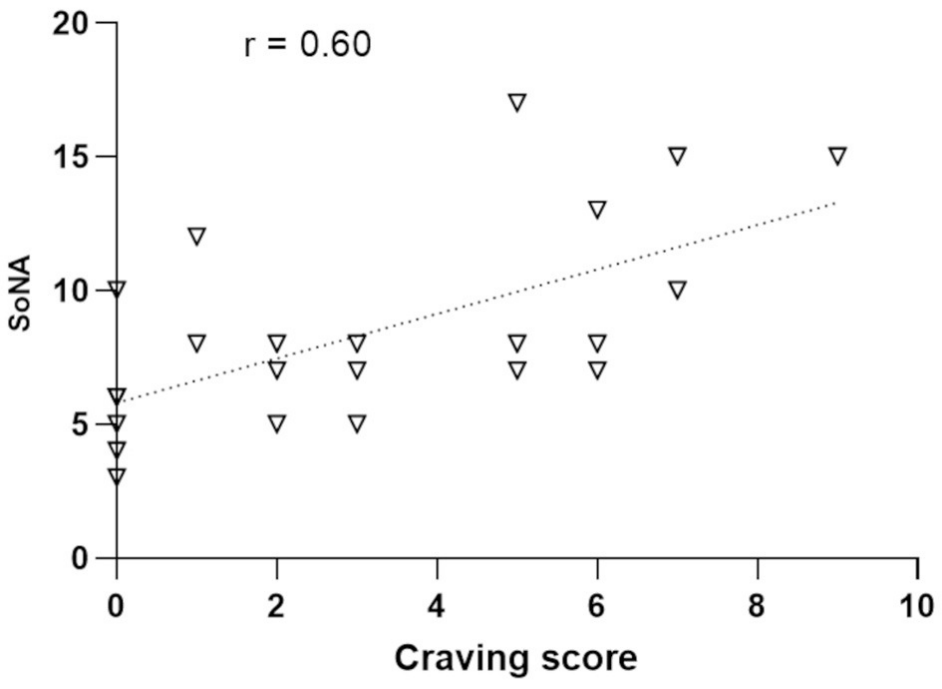
Correlational analysis between the craving scores measured after the walking session in the urban environment (abscissa) and Sense of Negative Agency (SoNA; ordinates).

In the City condition, a positive correlation (ρ = 0.60, *p* = 0.002) was found between craving and SoNA scores at the post-session time point (Figure 10). No significant correlations were found in the Nature condition.

## 4 Discussion

In our study, a 1-h Nature walk in the Venetian lagoon environment significantly decreased cravings compared to pre-walk values and compared to cravings after a 1-h City walk, with the latter significantly increased when compared to pre-walk values. These results confirm our hypothesis H1 and suggest that even brief exposure to natural environments may decrease craving not only in SUD patients but also in the general population, as investigated by previous studies (Versland and Rosenberg, 2007; Hamilton et al., 2013; Tesler et al., 2018; Martin et al., 2019). The same type of comparisons showed a significant decrease in total mood disturbances (POMS), and increased wellbeing (Flourishing Scale) and Sense of Positive Agency, confirming our hypotheses (H2, H3, and H5) and extending the results of previous studies (Shin et al., 2012; Tesler et al., 2018; Ochiai et al., 2020). Openness to the Future and Restorativeness measures showed a significant improvement after the Nature vs. City walk. This last point verifies our hypothesis H6: The lagoon environment chosen for the present study has been shown to exert a greater restorative effect than the urban environment in all subdimensions investigated; attracting attention and distracting participants from daily worries (subscales Fascination and Being away), being perceived as a space with meaning and being pleasantly explorable (subscales Coherence and Scope). Craving scores after a City walk positively correlated with POMS and a Sense of Negative Agency values and negatively correlated with the Flourishing scale values.

Participants were admitted to the residential facility at an average of 1 month before the start of the study. They were, therefore, already receiving the TAU for drug detoxification and rehabilitation, which included interventions for addiction and individual and group psychotherapy. These therapies could explain the low levels of craving at the pre-sessions time points (both for City and Nature conditions). Nevertheless, the Nature walk was able to further decrease craving. Our findings confirm that “nature experience”—such as a guided walk in a natural environment that includes elements (such as water, plants, and trees) known to exert effects on mental health—may improve mood, wellbeing, attention, stress relief, openness, and a sense of being active. Moreover, we also showed a specific effect on craving—a key symptom of SUD.

The correlational analysis did not show any association between craving and the other psychological measures (H7 to H10), despite the working hypothesis that the effects on mental functions might restore the affective, emotional, and cognitive unbalances underlying drug addiction. Therefore, we may hypothesize that the Nature walk under our conditions induced beneficial effects on both psychological and addictive measures but probably through different mechanisms. Although we did not investigate such underlying mechanisms, we could however hypothesize the involvement of processes that are affected by nature experience, such as those reported by mountain and forest studies (Kuo, 2015). In particular, a phenomenon that has been frequently observed is restoration. Restoration is the relaxed recovery of attention and cardiovascular parameters, that is, an effortless cognitive load, a positive initial affective response, or both. Bottom-up visual information (e.g., Hägerhäll et al., 2015; Kardan et al., 2015) activates affective and emotional processing, aligning with expectations and involves prefrontal cognitive functions (Joung et al., 2015), focusing attention and triggering endogenous reward expectations (Valtchanov and Ellard, 2015). The high restorativeness scores mentioned earlier (in particular, the Fascination subscale refers precisely to “the effortless and involuntary attention to the natural setting that is the key process to restore from mental fatigue”) suggest this possible interpretation.

We planned our study to show that the benefits of a natural environment experience were better than the daily experiences in the residence center. The residential center “Centro Soranzo” is in a former military facility that was recently redesigned with evidence-based and neuroarchitecture design features. These features of the space might presumably contribute to the mental health of the subjects and possibly help to lower craving values as assessed at the center before the walking sessions. Furthermore, craving values after the Nature walk were significantly lower, suggesting an additional benefit to the TAU effect on craving and the psychological measures.

As far as the comparison design is concerned, previous studies have investigated the effects of mountain hiking (indoor vs. outdoor activity) on the affective state (Niedermeier et al., 2017), or of forest therapy vs. mountain hiking on mental health (Huber et al., 2023). We also included as a comparator a walk in an urban area (Mestre city), which is not only characterized by contemporary architectural renovation but also by twenteeth-century buildings and degraded social housing. The comparison was not based on a specific hypothesis since the literature on the effects of exposure to urban context, already largely investigated, reported mixed results depending on specific features of the urban space (Hartig et al., 2003; Roe et al., 2013; White et al., 2013; Bratman et al., 2015). In fact, several reports showed the beneficial effects of nature vs. urban experience (e.g., Hartig et al., 2003), whereas other studies showed that green exposure in an urban setting may reduce stress (Roe et al., 2013), improve wellbeing (White et al., 2013), and enhance cognition (Bratman et al., 2015). In our study, the decrease in craving after the Nature walk was significantly different from the Urban after-walk measure, evidently due to the large craving increase in the latter group compared to the pre-walk assessment. Moreover, the increase in craving after the Urban walk was correlated to the impairment of mood, wellbeing, sense of autonomy, and optimism for the future. Similar data were obtained for emotions, mood, restorativeness, and subjective vitality when comparing a nature forest environment to a “building” control, with improvement and impairment in the indices of mental health, respectively (Bielinis et al., 2021). “Cue Reactivity” is the phenomenon that could underlie the observed effect in our participants. Cue reactivity is the response to salient information associated with rewards (Niaura et al., 1988; Rohsenow et al., 1991; Chiamulera, 2005). Not only discrete stimuli (i.e., cues) may be determinant factors of craving, but also the living space (the spatial context) may play a relevant role as a determinant factor (Conklin and Tiffany, 2002; Conklin, 2006; Conklin et al., 2008; Chiamulera et al., 2017). Our data, collected after the participants walked in an urban area where most of them used to live, that is an environment rich in conditioned stimuli and context associated with the substance, suggest that cue and context reactivity should be taken into consideration when evaluating residential program outcomes after patients' discharge. “*Re-naturing”* of urban spaces is a process that is recommended by several policies: integration of nature exposure into the planning and design of urban, architectural, and public spaces may change the value of the urban environment to a better, or at least less negative, place for mental health (Milgram, 1970; Bauduceau et al., 2015; Hartig and Kahn, 2016; Leloup et al., 2016; Astell-Burt et al., 2022) and possibly, as we suggest, for reducing relapse risk in former addicts. Future studies could also investigate more in depth about the role of urban environment features by testing the effect of different architectural and urban interventions and by identifying the elements that may induce a restorative response (and possibly, reduce cue reactivity). In this regard, a useful tool could be virtual reality (VR). Although the complexity of real-world investigations cannot be easily modeled in the laboratory, technologies such as VR may offer the possibility of ecologically and safely mimicking the effects of changing environmental context while maintaining rigorous control of experimental parameters (García-Rodríguez et al., 2012; Chiamulera et al., 2017, 2024; Spano et al., 2023; Theodorou et al., 2023).

Our study has some limitations. First, the participants were almost exclusively men with an average age of 40 years. Our data from a population of middle-aged male addicts cannot be inferred to a similar female sample, and, in general, to a younger population (Astell-Burt et al., 2013). Second, nature exposure limited to a single 1-h walk, without following sessions, does not allow us to make predictions about the relationship to exposure time or frequency dependency of the effect (White et al., 2019). Finally, some of the measures used do not have high internal consistency (especially SoAS).

Further studies are needed to assess the effects of changing Nature walk time durations to assess the relationship to response. Similarly, more frequent walks may help in assessing whether Nature walk effects persist or are transient and, maybe, whether a devaluation of the negative effects by Urban walk may take place after repetitive Urban walks (i.e., exposure to therapy-like effect).

## 5 Conclusion

In conclusion, our findings showed that experience in nature can reduce drug craving in SUD patients, and improve mood, wellbeing, openness to the future, and a sense of agency. In addition, an opposite pattern emerged after exposure to an urban environment, with craving scores associated with worsening mood, agency, and wellbeing. These findings suggest, on the one hand, the importance of considering the context when evaluating the outcomes of residential programs after patients are discharged and, on the other hand, when applying more efforts in evidence-based design of the urban environment, with both actions informed by multidisciplinary experimental and ecological research.

## Data availability statement

The raw data supporting the conclusions of this article will be made available by the authors, without undue reservation.

## Ethics statement

The studies involving humans were approved by the Ethics Committee of Centro Soranzo. The studies were conducted in accordance with the local legislation and institutional requirements. The participants provided their written informed consent to participate in this study.

## Author contributions

GB: Conceptualization, Formal analysis, Methodology, Writing – original draft. MS: Conceptualization, Investigation, Methodology, Writing – review & editing. AU: Writing – review & editing. IZ: Writing – review & editing, Investigation. MC: Conceptualization, Methodology, Project administration, Supervision, Writing – review & editing. CC: Conceptualization, Methodology, Project administration, Supervision, Writing – original draft.
