# Supplementary material for: Nature-based experience in Venetian lagoon: Effects on craving and wellbeing in addict residential inpatients
Source: Front Psychol. 2024 Jun 12;15:1356446. doi: 10.3389/fpsyg.2024.1356446 (PMC11202661; doi:10.3389/fpsyg.2024.1356446)
Supplement: Supplementary file 1 [file Table_1.DOCX]

Table S1. Mean and standard deviation of the measures in the three group

|  | Nature, Pre | Nature, Post | City, Pre | City, Post |
| --- | --- | --- | --- | --- |
| Craving (group 1) | 1.14 ± 1.95 | 0.50 ± 0.76 | 0.86 ± 1.46 | 2.00 ± 2.77 |
| Craving (group 2) | 2.00 ± 2.16 | 0.43 ± 0.79 | 1.13 ± 2.47 | 4.63 ± 2.88 |
| Craving (group 3) | 1.25 ± 2.05 | 1.13 ± 2.10 | 1.57 ± 1.62 | 2.75 ± 2.38 |
| POMS (group 1) | 20.13 ± 21.79 | 2.00 ± 15.18 | 19.29 ± 33.80 | 31.57 ± 37.66 |
| POMS (group 2) | 38.75 ± 47.87 | 1.75 ± 15.24 | 44.75 ± 63.28 | 60.88 ± 69.05 |
| POMS (group 3) | 35.50 ± 56.81 | 23.75 ± 53.67 | 42.50 ± 31.77 | 26.88 ± 29.49 |
| FS (group 1) | 29.00 ± 6.05 | 30.75 ± 4.56 | 27.71 ± 4.89 | 26.43 ± 6.95 |
| FS (group 2) | 25.33 ± 6.09 | 30.00 ± 4.60 | 24.88 ± 7.77 | 25.63 ± 7.05 |
| FS (group 3) | 25.63 ± 6.21 | 27.38 ± 6.63 | 23.00 ± 7.75 | 25.75 ± 5.04 |
| OFS (group 1) | 38.75 ± 6.82 | 37.63 ± 5.85 | 36.29 ± 6.02 | 31.14 ± 9.34 |
| OFS (group 2) | 34.75 ± 6.25 | 37.38 ± 4.84 | 31.50 ± 9.30 | 30.50 ± 9.86 |
| OFS (group 3) | 31.75 ± 8.12 | 34.38 ± 9.10 | 33.88 ± 6.79 | 33.13 ± 7.81 |
| SoPA (group 1) | 18.50 ± 4.99 | 20.29 ± 3.59 | 18.86 ± 3.85 | 17.86 ± 4.91 |
| SoPA (group 2) | 18.00 ± 5.13 | 20.75 ± 3.45 | 18.13 ± 4.12 | 16.13 ± 5.91 |
| SoPA (group 3) | 17.13 ± 7.30 | 20.00 ± 6.91 | 18.75 ± 5.12 | 19.13 ± 4.94 |
| SoNA (group 1) | 6.13 ± 2.95 | 6.86 ± 1.86 | 5.71 ± 2.98 | 7.71 ± 2.87 |
| SoNA (group 2) | 8.63 ± 2.77 | 7.25 ± 1.39 | 8.25 ± 3.73 | 9.63 ± 4.14 |
| SoNA (group 3) | 8.50 ± 4.21 | 7.88 ± 4.49 | 7.75 ± 2.55 | 7.88 ± 4.09 |
| FAS (group 1) |  | 6.71 ± 2.16 |  | 4.42 ± 3.06 |
| FAS (group 2) |  | 8.29 ± 1.79 |  | 2.21 ± 1.97 |
| FAS (group 3) |  | 6.54 ± 2.12 |  | 3.83 ± 2.24 |
| B-A (group 1) |  | 7.29 ± 1.52 |  | 3.67 ± 3.15 |
| B-A (group 2) |  | 8.00 ± 1.75 |  | 1.46 ± 1.63 |
| B-A (group 3) |  | 7.71 ± 1.48 |  | 2.00 ± 1.45 |
| COH (group 1) |  | 6.25 ± 1.99 |  | 4.04 ± 3.14 |
| COH (group 2) |  | 6.29 ± 1.23 |  | 3.46 ± 2.96 |
| COH (group 3) |  | 5.88 ± 1.81 |  | 4.08 ± 1.99 |
| SCO (group 1) |  | 5.63 ± 2.43 |  | 4.13 ± 2.12 |
| SCO (group 2) |  | 8.44 ± 1.32 |  | 2.75 ± 2.15 |
| SCO (group 3) |  | 8.13 ± 0.74 |  | 4.81 ± 2.22 |

Abbreviations: Nature: nature condition; City: city condition; Pre: pre-session measure; Post: post-session measure; POMS: Profile of Mood States; FS: Flourishing Scale; OFS: Openness to the Future Scale; SoPA: Sense of Positive Agency; SoNA: Sense of Negative Agency; FAS: Fascination; B-A: Being away; COH: Coherence; SCO: Scope
